# Hibernation in bats (Mammalia: Chiroptera) did not evolve through positive selection of leptin

**DOI:** 10.1002/ece3.4674

**Published:** 2018-11-28

**Authors:** Margaret E. Lazzeroni, Frank T. Burbrink, Nancy B. Simmons

**Affiliations:** ^1^ Richard Gilder Graduate School American Museum of Natural History New York New York; ^2^ Division of Vertebrate Zoology, Department of Herpetology American Museum of Natural History New York New York; ^3^ Division of Vertebrate Zoology, Department of Mammalogy American Museum of Natural History New York New York

**Keywords:** ancestral state reconstruction, bats, hibernation, homeothermy, leptin, thermoregulation, torpor

## Abstract

Temperature regulation is an indispensable physiological activity critical for animal survival. However, relatively little is known about the origin of thermoregulatory regimes in a phylogenetic context, or the genetic mechanisms driving the evolution of these regimes. Using bats as a study system, we examined the evolution of three thermoregulatory regimes (hibernation, daily heterothermy, and homeothermy) in relation to the evolution of leptin, a protein implicated in regulation of torpor bouts in mammals, including bats. A threshold model was used to test for a correlation between lineages with positively selected *lep,* the gene encoding leptin, and the thermoregulatory regimes of those lineages. Although evidence for episodic positive selection of *lep* was found, positive selection was not correlated with lineages of heterothermic bats, a finding that contradicts results from previous studies. Evidence from our ancestral state reconstructions suggests that the most recent common ancestor of bats used daily heterothermy and that the presence of hibernation is highly unlikely at this node. Hibernation likely evolved independently at least four times in bats—once in the common ancestor of Vespertilionidae and Molossidae, once in the clade containing Rhinolophidae and Rhinopomatidae, and again independently in the lineages leading to *Taphozous melanopogon* and *Mystacina tuberculata*. Our reconstructions revealed that thermoregulatory regimes never transitioned directly from hibernation to homeothermy, or the reverse, in the evolutionary history of bats. This, in addition to recent evidence that heterothermy is best described along a continuum, suggests that thermoregulatory regimes in mammals are best represented as an ordered continuous trait (homeothermy ← → daily torpor ← → hibernation) rather than as the three discrete regimes that evolve in an unordered fashion. These results have important implications for methodological approaches in future physiological and evolutionary research.

## 1 INTRODUCTION

In mammals, the evolution of endothermy—the use of metabolically produced heat to maintain *T*_B_ (body temperature)—has enabled survival of many different taxa across a variety of ecological niches and ecosystems (Fristoe et al., 2015; Hillenius & Ruben, 2004; Scholander, Hock, Walters, & Johnson, 1950). Homeothermy is defined by maintenance of a relatively constant *T*_B_ over time (Ivanov, 2006), and endothermy and homeothermy are frequently linked in mammals. Endothermic heterothermy, characterized by a combination of metabolically produced heat and the use of torpor—a physiological state characterized by the reduction in metabolism, and subsequently *T*_b_, below basal levels—has enabled the survival of mammalian taxa globally, especially in extremely cold climates, highly variable climates, or regions with periodic constraints on food and water availability (Geiser & Stawski, 2011; Geiser & Turbill, 2009; Tattersall et al., 2012). The duration of discrete torpor bouts is often used to define the two traditionally recognized forms of heterothermy in mammals: hibernation and daily heterothermy, also known as daily torpor (Geiser, 2004; Ruf & Geiser, 2015). Hibernators are capable of multiday torpor bouts and drastic Tb reduction, while daily heterotherms exclusively use shorter (<24 hr), shallower torpor bouts (Geiser, 2004; Ruf & Geiser, 2015). However, this binary categorization of heterothermy has been debated, with some authors arguing that hard boundaries between hibernation and daily torpor do not exist (Boyles et al., 2013; Canale, Levesque, & Lovegrove, 2012; Geiser & Ruf, 1995; Ruf & Geiser, 2015; van Breukelen & Martin, 2015). As new methods are developed, support for a continuum of heterothermic abilities has been growing but a consensus has not been reached (Boyles, Bennett, Mohammed, & Alagaili, 2017; Levesque, Nowack, & Stawski, 2016).

Although the common ancestor of mammals was traditionally presumed to be homeothermic (Crompton, Taylor, Jagger, & a, 1978), some recent authors have argued against this interpretation on physiological and/or behavioral grounds (Grigg, Beard, & Augee, 2004; Lovegrove, 2012). Grigg et al (2004) suggested that endothermic heterothermy may be ancestral to homeothermy in mammals because endothermic heterothermy would provide a reasonable intermediate along the transition from ectothermy to full endothermic homeothermy, a shift that would have required a many‐fold increase in metabolism (Geiser & Stawski, 2011).

Transitions to new thermoregulatory regimes over evolutionary time require physiological changes. Leptin, a hormone encoded by the *lep* gene, influences thermoregulation and satiety by signaling to the brain the degree of adiposity available for energy intake (Dodd et al., 2014; Enriori, Sinnayah, & Simonds, 2011; Kaiyala, Ogimoto, & Nelson, 2015; Zhang et al., 2011). Due to the role it plays in regulating satiety and metabolic activity, leptin concentrations may also be impacted by diet (Clarke & Connor, 2014; Harvey et al., 2000; Weigle et al., 2005). Leptin function is correlated with post‐hibernation weight gain in arctic ground squirrels (Boyer et al., 1997), pre‐hibernation weight gain in male woodchucks (Concannon, Levac, & Rawson, 2001), and feeding activity of at least one homeothermic bat (*Scotophilus heathii*; Srivastava & Krishna, 2008). Exon 3 of *lep* may be particularly important because it contains 29 amino acid variants with functional significance (Yuan et al., 2011). Some hibernating bats show structural alterations in exon 3 of *lep* that may cause leptin to become more physiologically active (He et al., 2010). Outside of the relationship leptin has with food intake and temperature regulation, leptin has a mechanistically independent function in initiating torpor bouts in some species (Stehling, Doring, & Ertl, 1996; Swoap, 2008). As part of a complex matrix of gene–gene interactions, sympathetic activation of white adipose tissue during fasting triggers a dramatic decrease in circulating leptin which is thought to initiate a torpor bout (Swoap, 2008). Entrance into torpor for Siberian hamsters is triggered by leptin (Freeman, Lewis, & Kauffman, 2004), and regulation of *T*_B_ is impacted by leptin in homeothermic rats (Stehling et al., 1996).

Bats (Chiroptera) represent a useful model for comparative evaluation of the evolutionary genomics of thermoregulatory regimes because they are a large monophyletic group with nearly 1,400 extant species, and show considerable variation in thermoregulatory regimes across the phylogeny including homeothermy, daily torpor, and hibernation (Lyman, 1970; Simmons, 2005; Stawski, Willis, & Geiser, 2014). Bats are also among the most diverse mammalian clades with respect to diet, with various lineages specialized as insectivores, carnivores, frugivores, nectarivores, omnivores, and even sanguinivores (Fenton & Simmons., 2015; Hill & Smith., 1984).

Consistent with studies on other mammals, leptin in bats influences torpor abilities, feeding activity, and energy balance (Banerjee, Udin, & Krishna, 2011; He et al., 2010; Kronfeld‐Schor, Richardson, & Silvia, 2000; Srivastava & Krishna, 2008; Yuan et al., 2011; Zhu et al., 2014). Ancestral state reconstruction based on the evolution of the *lep* gene (Yuan et al., 2011) and circumstantial evidence based on the abundance of tropical and subtropical heterothermic bats (Cory Toussaint, McKechnie, & Merwe, 2010; Geiser & Stawski, 2011; Liu & Karasov, 2011; Stawski, Turbill, & Geiser, 2009; Turbill, Law, & Geiser, 2003) have been used to suggest that the most recent common ancestor of bats was heterothermic. However, it is unclear if this ancestor used hibernation or daily torpor.

To better understand the unique evolutionary history of thermoregulation, we examined the relationship between thermoregulatory regime of extant taxa and selection on *lep* exon 3. Yuan et al. (2011) found that leptin has undergone positive selection in heterothermic bat lineages, and therefore associated it with the evolution of torpor. However, by using a *lep* exon 3 gene tree instead of a species tree, Yuan et al. (2011) effectively treated two nonindependent variables (branch length and *lep* selection) as independent. Coalescence theory suggests that the use of gene trees to calculate ω, a measure of positive selection, may bias results because gene trees do not necessarily mirror species trees, and may have alternative branching patterns and lengths compared to the species tree (Diekmann & Pereira‐Leal, 2016). To reexamine the findings of Yuan et al. (2011), we hypothesized that *lep* exon 3 has undergone episodic positive selection in heterothermic lineages. Episodic positive selection is defined as positive selection which occurs in a subset of lineages. We tested this hypothesis in the context of a species tree for relevant bat taxa. We also reconstructed the ancestral thermoregulatory regimes of bats with revised species‐level data compared to previous family‐level analyses (Yuan et al., 2011). By evaluating the number of transitions between regimes across the phylogeny through stochastic character mapping, we also evaluated the validity of categorizing thermoregulatory regimes as three discrete character states. Finally, to address other known effects of leptin, we tested for correlation between lineages with positively selected *lep* exon 3 and the diets of those lineages.

## 2 MATERIALS AND METHODS

### 2.1 Taxon sampling

To test for positive selection of *lep* exon 3, 31 species of bats with known thermoregulatory regimes were sampled for the *lep* exon 3 gene, including representatives from nine families (Table 1). Thirteen of these species hibernate, five use daily torpor, and 13 are homeothermic (Table 1). *Lep* exon 3 sequences for 27 of these species were downloaded from GenBank, and the other four, *Pteropus hypomelanus, Nyctimene major, Macroglossus minimus,* and *Syconyteris australis,* were sequenced in the Sackler Institute for Comparative Genomics. *Lep* exon 3 sequences for three outgroups were also included, *Homo sapiens* (human), *Capra hircus* (goat), and *Galeopterus variegatus* (Sunda colugo; Table 1). Species‐specific data on thermoregulatory regimes and dietary preferences were taken from the primary literature (Table 1, dietary preferences only listed for species in this dataset). The resulting dataset included 34 taxa, which we used for analyses of positive selection of *lep* exon 3 and models evaluating the relationship between positive selection of *lep* exon 3 and thermoregulatory strategies or diet.

**Table 1:** Overview of the thermoregulatory regimes and diet of bat species included in this study

Taxa	TR	TR reference	Diet	Diet reference	Accession #	Loan Institution: ID
Bats						
*Anoura geoffroyi* a	Daily Torpor^b^	Audet and Thomas (1997), Avila‐Flores and Medellín (2004), Stawski et al. (2014)	Insectivorous	Baker, Jones, and Carter (1976), Ortega and Alarcón‐D (2008)	GU230833	
*Carollia perspicillata*	Daily Torpor	Audet and Thomas (1997)				
*Dermanura gnoma* a	Daily Torpor^b^	Audet and Thomas (1997), Avila‐Flores and Medellín (2004), Stawski et al. (2014)	Frugivorous	Solari et al. (2009), Rojas, Vale, Ferrero, and Navarro, (2011)	GU230832	
*Erophylla bombifrons*	Daily Torpor	Rodriguez‐Duran (1995)				
*Eumops perotis*	Daily Torpor	Leitner (1966)				
*Glossophaga soricina*	Daily Torpor	Rasweiler (1973)				
*Lasiurus seminolus*	Daily Torpor	Genoud (1993)				
*Macroglossus minimus* a	Daily Torpor	Bartels, Law, and Geiser (1998), Geiser (2006)	Nectarivorous	Nowak (1994), Hollar and Springer (1997)		AMCC: CEF801
*Megaloglossus woermanni*	Daily Torpor	Kulzer and Storf (1980)				
*Monophyllus redmani*	Daily Torpor	Rodriguez‐Duran (1995)				
*Mops condylurus*	Daily Torpor	Maloney, Bronner, and Buffenstein (1999)				
*Myotis daubentonii*	Daily Torpor	Dietz and Kalko (2006)				
*Nycteris thebaica*	Daily Torpor	Cory Toussaint and McKechnie (2012)				
*Nyctimene albiventer*	Daily Torpor	Bartholomew, Dawson, and Lasiewski (1970)				
*Nyctimene robinsoni*	Daily Torpor	Geiser (2006)				
*Peropteryx macrotis*	Daily Torpor	Genoud, Bonaccorso, and Anends (1990)				
*Pternotus davyi*	Daily Torpor	Czenze and Dunbar (2017)				
*Rhinolophus megaphyllus*	Daily Torpor	Young (2001)				
*Scotophilus dinganii*	Daily Torpor	Jacobs, Kelly, Mason, and Stoffberg (2007)				
*Sturnira lilium*	Daily Torpor	Audet and Thomas (1997)				
*Syconycteris australis* a	Daily Torpor	Geiser (2006)	Nectarivorous	Altringham (1996), Hollar and Springer (1997), Courts (1998)		Smithsonian: 585524
*Tadarida teniotis* a	Daily Torpor	Arlettaz et al. (2000)	Insectivorous	Freeman (1979), Rydell and Arlettaz (1994), Whitaker and Karataş (2009)	GU230839	
*Taphozous australis*	Daily Torpor	Kulzer, Nelson, McKean, and Möhres (1970), Geiser (2006)				
*Barbastella barbastellus*	Hibernation	Pohl (1961), Russo et al. (2017)				
*Chaerephon plicatus* a	Hibernation	Vivier and Van Der Merwe (2007), Yuan et al. (2011)	Insectivorous	Freeman (1979), Bohmann et al. (2011), Kusuminda and Yapa (2017)	GU230836	
*Chalinolobus gouldii*	Hibernation	Stawski and Currie (2016)				
*Eptesicus fuscus* a	Hibernation	Brack (2007)	Insectivorous	Agosta (2002)	NW_007370746	
*Hipposideros armiger* a	Hibernation	Liu and Karasov (2011)	Insectivorous	Whitaker and Karataş (2009), Weterings, Wardenaar, Dunn, and Umponstira (2015)	NW_017731447	
*Lasionycteris noctivagans*	Hibernation	Izor (1979)				
*Lasiurus borealis*	Hibernation	Dunbar and Tomasi (2006)				
*Lasiurus cinereus*	Hibernation	Cryan (2003), Willis, Brigham, and Geiser (2006)				
*Miniopterus fuliginosus* a	Hibernation	Kimura and Uchida (1983), Yuan et al. (2011)	Insectivorous	Hu, Wei, Zhu, Wang, and Zhang (2011)	GU230844	
*Miniopterus natalensis* a	Hibernation	Van Der Merwe (1973)	Insectivorous	Naidoo, Mackey, and , Schoeman MC, (2011)	NW_015504548	
*Miniopterus schreibersii*	Hibernation	Kulzer et al. (1970), Geiser (2006)				
*Myotis adversus*	Hibernation	Kulzer et al. (1970)				
*Myotis brandtii* a	Hibernation	Villanueva‐Cañas et al. (2014)	Insectivorous	Vaughan (1997), Whitaker and Karataş (2009)	NW_005360677	
*Myotis davidii* a	Hibernation	Villanueva‐Cañas et al. (2014)	Insectivorous	Zhang et al. (2013)	NW_006296405	
*Myotis leibii*	Hibernation	Best and Jennings (1997)				
*Myotis lucifugus* a	Hibernation	Brack (2007)	Insectivorous	Belwood and Fenton (1976)	NW_005871121	
*Myotis myotis*	Hibernation	Pohl (1961), Harmata (1987), Koteja, Jurczyszyn, and Woloszyn (2001)				
*Myotis nattereri*	Hibernation	Hope and Jones (2012)				
*Myotis ricketti* a	Hibernation	Zhang et al. (2014)	Piscivorous	Ma et al. (2003)	GU230846	
*Myotis septentrionalis*	Hibernation	Brack (2007)				
*Myotis sodalis*	Hibernation	Brack (2007)				
*Myotis velifer*	Hibernation	Tinkle and Patterson (1965), Riedesel and Williams (1976)				
*Myotis vivesi*	Hibernation	Salinas, Herrera, Flores‐Martínez, and Johnston (2014)				
*Nyctalus noctula*	Hibernation	Ransome (1990), Arlettaz et al. (2000)				
*Mystacina tuberculata*	Hibernation	Czenze, Brigham, Hickey, and Parsons (2017a)				
*Nyctophilus geoffroyi*	Hibernation	Turbill et al. (2003)				
*Nyctophilus gouldi*	Hibernation	Turbill et al. (2003)				
*Pipistrellus pipistrellus*	Hibernation	Kayser (1964), Kulzer (1965)				
*Plecotus auritus*	Hibernation	Eisentraut (1956)				
*Rhinolophus ferrumequinum* a	Hibernation	Park, Jones, and Ransome (1999); Chen, Yuan, and Sun (2008)	Insectivorous	Vaughan (1997), Whitaker and Karataş (2009)	GU230845	
*Rhinolophus hipposideros*	Hibernation	Harmata (1987)				
*Rhinopoma microphyllum* a	Hibernation	Levin and Kronfeld‐schor (2012), Stawski et al. (2014)	Insectivorous	Sharifi and Hemmati (2002)	GU230830	
*Scotophilus heathii* a	Hibernation	Rashid, Irfan, Nadeem, and Shabbir (2016)	Insectivorous	Jacobs, Eick, Schoeman, and Matthee (2006)	GU230843	
*Tadarida aegyptiaca*	Hibernation	Geiser and Stawski (2011)				
*Tadarida brasiliensis*	Hibernation	Herreid (1963), Herreid and Schmidt‐Nielsen (1966)				
*Taphozous melanopogon* a	Hibernation	Kulzer (1965)	Insectivorous	Hu et al. (2011), Weterings et al. (2015)	GU230842	
*Carollia brevicauda* a	Homeothermy	Avila‐Flores and Medellín (2004)	Frugivorous	Fleming (1991)	GU230829	
*Cynopterus sphinx* a	Homeothermy^b^	Banerjee, Meenakumari, and Krishna (2007), Stawski et al. (2014)	Frugivorous	Ruby, Nathan, Balasingh, and Kunz (2000)	GU230842	
*Dobsonia viridis* a	Homeothermy^b^	Stawski et al. (2014)	Frugivorous	Bonaccorso, Winkelmann, Dumont, and Bat (2002)	GU230840	
*Eidolon helvum* a	Homeothermy	Zaidan (1980)	Frugivorous	Richter and Cumming (2006)	GU230838	
*Eonycteris spelaea* a	Homeothermy	Krutzsch (1979)	Nectarivorous	Fenton (1990), Bumrungsri et al. (2013)	GU230848	
*Macroderma gigas*	Homeothermy	Lyman (1970)				
*Mormoops blainvilli*	Homeothermy	Bonaccorso et al. (1992), Rodriguez‐Duran (1995)				
*Nyctimene major* a	Homeothermy	Bartholomew et al. (1970)	Frugivorous	Freeman (1995)		AMCC: PRS2767
*Pteronotus parnelli* a	Homeothermy	Bonaccorso et al. (1992); Stawski et al. (2014)	Insectivorous	Fenton (1990), Brigham (1991), Rojas et al. (2011)	GU230831	
*Pteronotus personatus*	Homeothermy	Bonaccorso et al. (1992), Stawski et al. (2014)				
*Pteronotus quadridens*	Homeothermy	Rodriguez‐Duran (1995)				
*Pteropus alecto* a	Homeothermy^b^	McNab and Bonaccorso (2001)	Herbivorous	Zhang et al. (2013)	NW_006434839	
*Pteropus giganteus* a	Homeothermy	Kulzer (1965)	Frugivorous	Nowak (1994), Vaughan (1997)	GU230837	
*Pteropus hypomelanus* a	Homeothermy	Ochoa‐Acuña and Kunz (1999)	Frugivorous	Heard and Whittier (1997)		AMCC: PER 1
*Pteropus vampyrus* a	Homeothermy	McNab and Armstrong (2001)	Herbivorous	Stier and Mildenstein (2005)	NW_011888814	
*Rousettus aegyptiacus* a	Homeothermy	Noll (1979)	Frugivorous	Korine, Izhaki, and Arad (1999)	NW_015494499	
*Rousettus leschenaultii* a	Homeothermy^b^	Noll (1979), Stawski et al. (2014)	Frugivorous	Raghuram, Thangadurai, and Gopukumar (2009)	GU230847	
Outgroups						
*Capra hircus* a	Homeothermy	Kaciuba‐Usciz.xl;lexo, Jessen, Feistkorn, and Brzezinska (1987)	Folivorous	Genin and Pijoan (1993)	AM114397	
*Galeopterus variegatus* a	Homeothermy		Herbivorous	Dzulhelmi and Abdullah (2009)	NW_007735418	
*Homo sapiens* a	Homeothermy	Mekjavic and Eiken (2006)	Omnivorous		D63519	

Notes

TR: thermoregulatory regime.

a Included in the dataset for the tests of positive selection of *lep* exon 3.

b Species‐specific thermoregulatory regime is undocumented in the literature. The most likely thermoregulatory regime was inferred based on associated literature and family‐level summaries of thermal physiology (Stawski et al., 2014).

A larger dataset including 76 bat taxa and three outgroups was used for the ancestral state reconstruction of thermoregulatory regimes. Here, we were not limited by the requirement of having leptin sequences, which allowed for greater coverage of the phylogeny. Species were selected for the ancestral state reconstruction based on availability of data describing their thermoregulatory regime and presence in the published phylogeny that was used in the analyses (i.e., Amador, Arévalo, & Almeida, 2016). Data on the thermoregulatory regime of each species were taken from the primary literature by searching databases (Google Scholar and Web of Science) between the dates of September 2015 and August 2018, using keywords “bats,” “thermoregulation,” “thermoregulatory regimes,” “temperature regulation,” “hibernation,” “torpor,” “daily torpor,” “daily heterothermy,” “heterothermy,” “homeothermy,” “metabolism,” and “Chiroptera.” These data are summarized in Table 1.

DNA was extracted and *lep* exon 3 was amplified and sequenced for four bat species for the tests for positive selection—*Pteronotus hypomelanus, Nyctimene major, Macroglossus minimus,* and *Syconycteris australis*. All laboratory work was conducted in the Sackler Institute for Comparative Genomics. DNA was extracted from either wing punches or tissues of museum specimens (Table 1) using the Qiagen DNeasy Blood and Tissue Extraction Kit. In order to amplify *lep* exon 3 in these species, a primer pair (F1—AGAAGGGAGGGAGGACTCAAC, R1—GCTTCAGCACCCAGGGCTG) was developed on the flanking region of the consensus sequence from a multiple alignment of published *lep* sequences from *Rousettus leschenaultii, Pteronotus giganteus, Eonycteris spelaea, Eidolon helvum, Dobsonia viridis,* and *Cynopterus sphinx* which was made by eye in Geneious (Kearse et al., 2012).

The polymerase chain reaction (PCR) was carried out using illustra^TM^ puReTaq Ready‐To‐Go PCR Beads Kit. Amplification was performed in a 25 µl reaction volume. This consisted of 20.7 µl nuclease‐free water, 0.3 µl bovine serum albumin, 1 µl 10× solution of forward primer, 1 µl 10× solution of reverse primer, 2 µl template, and one bead containing recombinant puReTaq DNA polymerase. PCR conditions were as follows: an initial denaturation phase at 95°C for 5 min, 25 cycles with a denaturation phase at 95°C for 30 s, an annealing phase at 57°C for 30 s, and an extension phase at 72°C for 45 s. The wells were then stored in a refrigerator at 4°C. PCR products were purified with AgenCourt AMPure XP. PCR products were sequenced using Sanger sequencing (Smith & Hood, 1987) following protocol from the BigDye® Terminator v3.1 Cycle Sequencing Kit.

Sequences were assembled and edited within Geneious (Kearse et al., 2012). Ends of forward and reverse sequences were trimmed with an error set to 0.01. Trimmed regions were ignored and forward and reverse sequences were assembled using de novo assembly, as outlined by the Geneious manual (Biomatters, 2017). The forward and reverse sequences of *lep* for *Nyctimene major* did not assemble through de novo assembly. Therefore, these were mapped to the reference sequence from which the primers were built. Reads were then manually edited to maximize the coverage and identity between the forward and reverse sequences. *Lep* exon 3 was trimmed to the open reading frame (ORF), and stop codons were removed from the tail. ORFs from all species in the dataset were then aligned by eye in Geneious (Kearse et al., 2012).

### 2.2 Branch–sites under positive selection

To identify lineages that have experienced episodic positive selection of *lep* exon 3, we ran a mixed‐effects model of evolution (MEME) to detect a subset of branch–sites under episodic positive selection (Murrell et al., 2012). MEME uses a fixed‐effect model to explain the distribution and variation of ω across sites, and a random‐effects model to explain variation in the distribution of *ω* across branches (Kosakovsky Pond et al., 2011; Murrell et al., 2012; Nielsen & Yang, 1998). MEME was used here because other tests, which usually average *ω* over branches and sites, often miss positive selection when it occurs in a subset of branch–sites (Yang & Nielsen, 2002). When evaluating if a branch–site is under positive selection, MEME considers the specific model of molecular evolution, which here was the TRN93 model (Tamura & Nei, 1993), differences in codon frequencies, and *ω*.

The test for branch–sites under episodic positive selection, MEME, was performed within the DataMonkey web server (Delport, Poon, Frost, & Kosakovsky Pond, 2010) using the aligned *lep* exon 3 sequences, the automatic substitution model selection tool, and a user‐specified tree from Amador et al. (2016). This 807 taxa phylogeny was calibrated using 44 key fossils, inferred using nine nuclear and mitochondrial genes, and shows support for the majority of currently recognized bat clades (Amador et al., 2016). The Amador et al. (2016) tree was chosen for this study because it represents the most genus‐ and species‐level diversity, 90% and 64%, respectively, compared to other phylogenies (Amador et al., 2016). The tree was pruned using the ape package in R (Paradis, Claude, & Strimmer, 2004) to only include the 31 bats and three outgroup taxa in our dataset. The model of molecular evolution that best fit these data was determined to be the TRN93 model (Tamura & Nei, 1993) using the automatic function available in DataMonkey (Delport et al., 2010).

The significance threshold was set to 0.1 and a log‐ratio test (LRT) was performed, comparing the alternative model to the null model. The alternative model allows for positive selection in a subset of branch–sites, while the null model does not allow for positive selection in a subset of branch–sites. For each branch, an empirical Bayes factor (EBF) for having *ω* > 1 was calculated with an associated posterior probability.

Mixed‐effects model of evolution only detects branch–sites under episodic positive selection, not lineages with gene‐wide positive selection. Therefore, to confirm that *lep* exon 3 is indeed under positive selection in the branches detected by MEME, we tested for gene‐wide episodic positive selection using BUSTED, a branch–site unrestricted statistical test for episodic positive selection (Murrell et al., 2015). BUSTED uses a LRT to detect evidence of episodic positive selection, when the rate of non‐synonymous to synonymous substitutions at branch–sites is transiently greater in the foreground branches compared to background (Murrell et al., 2015). Foreground branches are the lineages hypothesized to be under positive selection, and the background branches are all other branches in the phylogeny. This model assumes that the gene evolves under the general time reversal model (Tavaré, 1986).

We also used BUSTED to test for episodic positive selection by binning branch–sites into three *ω* categories representative of either purifying, neutral, or positive selection. Purifying selection is defined by having a *ω* < 1, indicating that there is strong selection to maintain the sequence over evolutionary time. Neutral selection is defined by having a *ω* approximately equal to 1, indicating that there is neither strong selection for the maintenance of a sequence over time nor selection for changes to that sequence. In the unconstrained model, both foreground and background branches can evolve under positive selection. In the null models, neither foreground nor background branches are allowed to evolve through positive selection. This analysis required, as input, the same alignment of *lep* exon 3 sequences used in the MEME analysis, and the pruned, user‐specified phylogeny from Amador et al. (2016).

**Figure 1:**
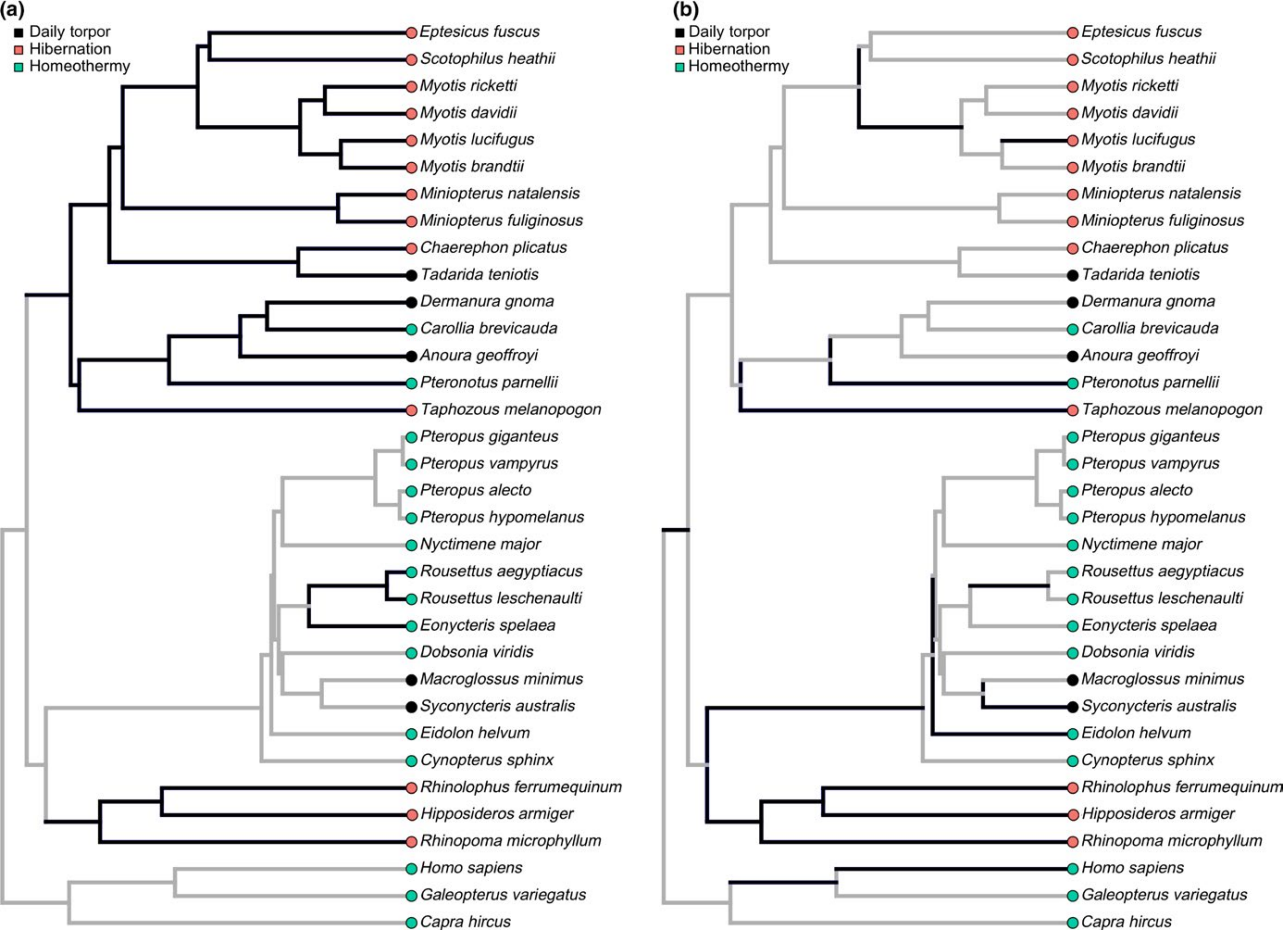
BUSTED hypotheses tests for positive selection of lep exon 3 across bats with different thermoregulatory regimes (black = daily torpor; pink = hibernation; green = homeothermy). Topology of tree generated from Amador et al. (2016). Bolded lineages represent the lineages tested for positive selection (foreground branches). Gray lineages represent the background. (a) Foreground branches correspond with those found to have evidence of positive selection by Yuan et al. (2011). (b) Foreground branches represent lineages detected by MEME to have evidence of positive selection

Two analyses to test for gene‐wide positive selection were performed. In the first analysis, we tested for selection along branches where evidence for positive selection was previously detected (Figure 1a; Yuan et al., 2011). In the second analysis, we selected foreground branches based on lineages with evidence for positive selection from the MEME results (Figure 1b). Here, branches were considered foreground if they met the following criteria based on the MEME output: A branch had at least one codon site with an EBF > 3 for having *ω* > 1, and a posterior probability >0.25 for having *ω* > 1. These criteria conform with the guidelines for interpreting EBF values from Kass and Raftery (1995). In addition to the LRT calculation, Akaike information criteria (AIC) scores were calculated. AIC statistically quantifies the quality of each model by considering the optimum log likelihood (l) and the number of parameters (p) (AIC = −2l + 2p), enabling model comparison.

### 2.3 Correlation between positive selection and phenotypic traits

To determine whether evolution of *lep* exon 3 is correlated with evolution of thermoregulatory regimes, we ran a threshold model (Felsenstein, 2012). Here, a discrete trait is presumed to change state when an underlying variable, the liability, crosses a certain threshold (Felsenstein, 2005, 2012 ). This liability is assumed to have a multivariate normal distribution and to evolve under Brownian motion (Felsenstein, 2005, 2012 ). Contrasting the commonly used continuous‐time Markov (Mkn) model of discrete character evolution (Lewis, 2001; Pagel, 1994), character states under the threshold model are inherently ordered and the evolution of discrete character states is not memoryless (Felsenstein, 2005, 2012 ); the character state at one node is influenced by the character state at previous nodes. We categorized thermoregulatory regimes into three states: hibernation, daily torpor, and homeothermy. The presence (1) or absence (0) of positive selection in *lep* exon 3 served as the liability. Species were considered to have undergone positive selection if *lep* exon 3 was determined to be under positive selection per the results from MEME and BUSTED. Only data for the terminal branches were considered, as character states at internal nodes are necessarily unknown.

Parameters for the model were estimated with a Bayesian Markov chain Monte Carlo (MCMC) approach using the threshBayes function in R, with default priors and liabilities (Revell, 2012). We ran the chain for 3 million generations, thinned to 1,000 samples per chain, to account for autocorrelation, and discarded the first 500,000 as burn‐in. To quantify the relationship between *lep* evolution and TR, a correlation coefficient was calculated between the liability and the thermoregulatory regime. Finally, the highest posterior densities (HPDs) were estimated from the correlation coefficients to determine whether the correlation between traits statistically differed from 0, indicating a statistically significant relationship between the two variables. To test the alternative hypothesis that *lep* exon 3 evolution is correlated with diet, we used the same methods as above but used diet as the discrete trait evolving under the liability.

### 2.4 Ancestral state reconstruction of thermoregulatory regimes

To model the ancestral thermoregulatory regimes of bats, we first used the Mkn model of discrete character evolution (Lewis, 2001; Pagel, 1994) with the states hibernation, daily torpor, and homeothermy. It was important to test which transition rate matrix best described the data. Transition matrices describe the rate of transitioning from state *i* to state *j*. Here, we tested the data to fit one of three transition matrices: (a) equal transitions between all states (equal rates), (b) different transition rates between, but not among, pairs of states (symmetric), and (c) different transition rates between and among pairs of states (all rates different). We also tested each transition matrix under different transformations to determine whether transition rates varied overtime. We ran these tests for 100 iterations within the fitDiscrete function in the *geiger* package in R (Harmon, Weir, & Brock, 2008). The model with the lowest weighted AIC score was subsequently chosen to run the ancestral state reconstruction. The symmetric model under a kappa transformation had the best fit to these data. This suggests that, given our data, transition rates vary over time depending on the number of speciation events between two species and that the transition rate between one pair of character states is identical in the forward and reverse but pairs of states can have different transition rates. No transformations were indicated by the data, indicating that the transition rate does not vary over time.

**Figure 2:**
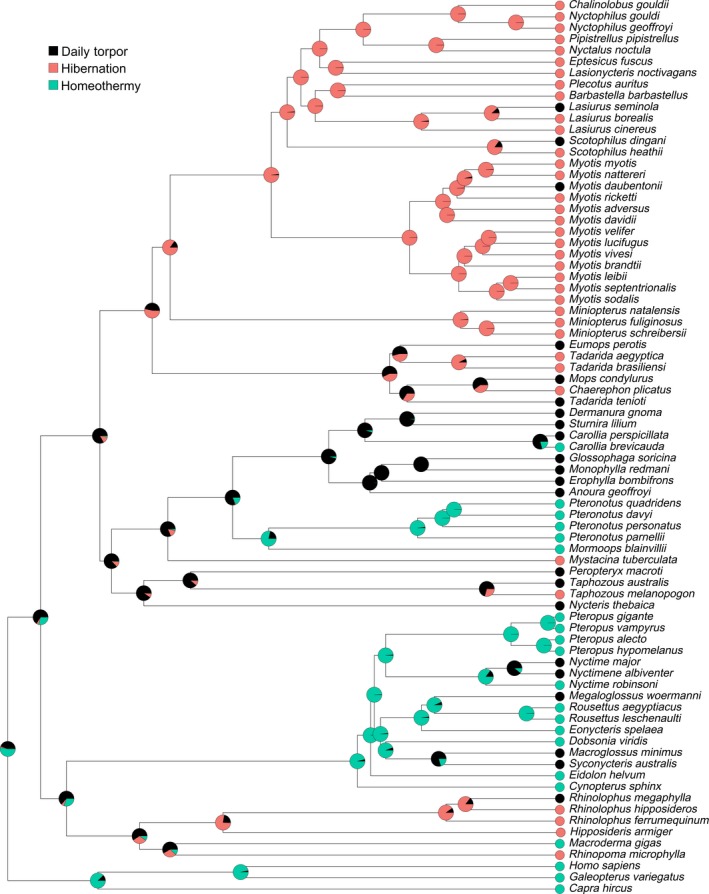
Ancestral state reconstruction under the Mkn model. Topology of tree generated from Amador et al. (2016). Pie charts represent the marginal likelihoods of each thermoregulatory regime at a given node

Given these parameters, we ran an ancestral state reconstruction using the Ace function from the *ape* package in R (Figure 2) with maximum likelihood estimation to obtain probabilities of states at interior nodes (Paradis et al., 2004). Stochastic character mapping (Bollback, 2006; Huelsenbeck, Nielsen, & Bollback, 2003) was also used to estimate states at interior nodes and to determine how well the chosen parameters matched the real data. Stochastic character maps were built using the make.simmap function in the *phytools* package in R (Revell, 2012). Fifty thousand simulations were run using the parameters described previously. A Q–Q plot was generated to compare the Mkn model to the stochastic character map to evaluate the goodness of fit. The stochastic character map was then used to quantify the number of transitions between states across simulations.

## 3 RESULTS

### 3.1 Data collection

We obtained new *lep* sequences for four bats, *Pteropus hypomelanus* (484 bp), *Nyctimene major* (487 bp), *Macroglossus minimus* (788 bp), and *Syconyteris australis* (489 bp). These were aligned to the other sequences in the dataset. Once aligned, sequences were trimmed to the coding region of *lep* exon 3 at 357 bp across 34 taxa.

### 3.2 Measuring positive selection

From the MEME results, evidence for positive selection was detected in 15 branches (Table 2) over a total of 10 codons (Table 3). When these lineages were selected as the foreground branches in the BUSTED analyses, evidence for episodic positive selection was detected (*p* = 0.009; Figure 1b). Positive selection was not detected when the foreground branches were selected according to those previously found by Yuan et al. (2011) to be under positive selection (*p* = 0.220).

**Table 2:** Branch–Sites with evidence of positive selection in *lep* exon 3

Branch	TR	Sites	EBF	Posterior probability
Node 3	—a	75	210.2	1
Node 8	—a	7	15.3	0.56
Node 10	—a	5	>1,000	1
		6	>1,000	1
		7	11.8	0.5
		75	15	0.86
		108	46.2	0.39
Node 13	—a	75	209	1
Node 26	—a	78	319.6	0.99
		86	42.1	1
Node 57	—a	86	436.3	1
*Eidolon helvum*	Homeothermic	86	>1,000	1
*Hipposideros armiger*	Hibernation	7	23.8	0.67
		75	48.4	0.95
		78	3.6	0.61
		108	529.1	0.88
*Homo sapiens*	Homeothermic	91	>1,000	1
*Myotis lucifugus*	Hibernation	6	>1,000	1
*Pteronotus parnellii*	Homeothermic	4	>1,000	1
		56	>1,000	1
		86	23.2	1
*Rhinolophus ferrumequinum*	Hibernation	7	17.3	0.59
		78	3.6	0.61
		108	206.5	0.74
*Rhinopoma microphyllum*	Hibernation	4	>1,000	1
		7	36.2	0.75
		78	234	0.99
*Syconycteris australis*	Daily Torpor	86	>1,000	1
*Taphozous melanopogon*	Hibernation	6	>1,000	1
		75	>1,000	1

Note

EBF: empirical Bayes factor for having *ω* > 1; Posterior: posterior probability; TR: thermoregulatory regime.

a Not Applicable.

**Table 3:** *Lep* exon 3 codons with evidence of positive selection in bats

Codon	*α*	Unconstrained *β*+	*ω*	*p*‐Value
4	0	7.21	∞	0.03
5	2.04	48.00	23.53	0.04
6	0	5.64	∞	0.03
7	0.56	43.45	77.59	0.05
56	0	207.15	∞	0.01
75	0	5.50	∞	0.02
78	0	5.84	∞	0.07
86	0	1.37	∞	0.08
91	0.66	11.68	17.70	0.06
108	0	38.31	∞	<0.001

Note

*α*: maximum likelihood estimation (MLE) of synonymous rate; *β*+: unconstrained MLE of non–synonymous rate.

### 3.3 Correlation between positive selection and phenotypic traits

No relationship was found between thermoregulatory regimes and lineages with positive selection of *lep* exon 3 (95% HPD = −0.271 to 0.600), or between positively selected lineages and diet (95% HPD = −0.222 to 0.692).

### 3.4 Ancestral state reconstruction

**Figure 3:**
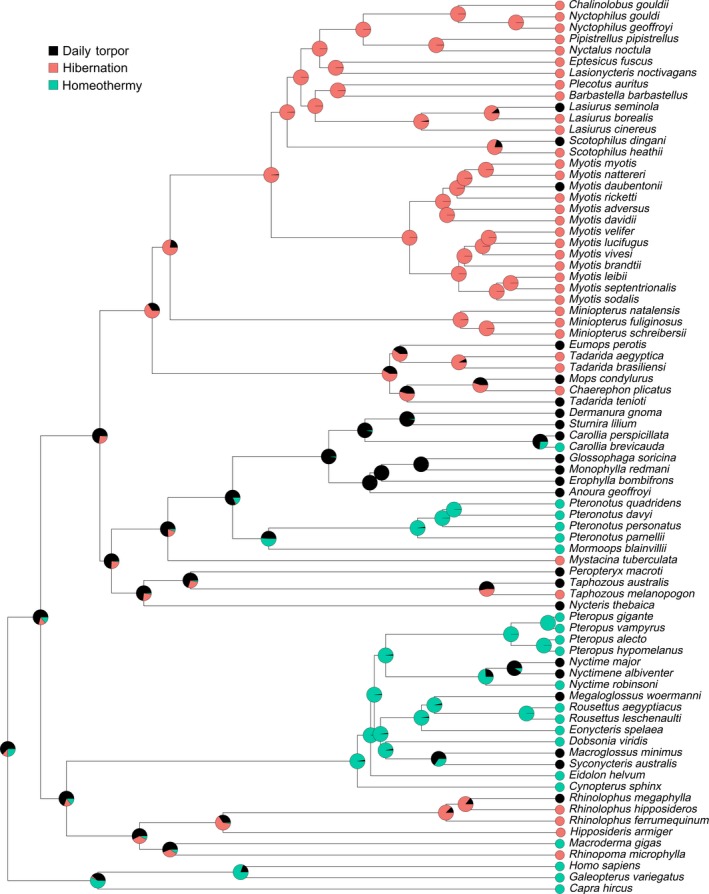
Ancestral state reconstruction from stochastic character mapping. Topology of tree generated from Amador et al. (2016). Pie charts represent the posterior probability of each thermoregulatory regime at a given node

The scaled likelihoods from the Mkn model (Supporting Information Table S1) and the posterior probabilities from stochastic character mapping were highly correlated (*ρ* = 0.985, *p* = 2.2 × 10^−16^; Figures 2 and 3). The state of the most recent common ancestor of bats could not be fully resolved in either model (Daily Torpor, logL = 0.660, posterior = 0.714; Hibernation, logL = 0.005, posterior = 0.154; Homeothermy, logL = 0.289 posterior = 0.131). In the Mkn model (Figure 2), the transition rate between daily torpor and hibernation was similar to that between daily torpor and homeothermy (MLE = 0.054 ± 0.012 and MLE = 0.052 ± 0.014, respectively), while no transitions were found between hibernation and homeothermy. Across the 50,000 stochastic simulations, an average of 33 state changes occurred per tree (summarized in Figure 3). Over each tree, an average of eight transitions occurred from daily torpor to hibernation, and 10 transitions occurred from hibernation to daily torpor. An average of eight transitions occurred from daily torpor to homeothermy, and seven transitions from homeothermy to daily torpor. Transitions between hibernation and homeothermy, in either direction, never occurred across all simulations. Across all regimes and simulations, the mean proportion of time spent in each state was 0.308, 0.436, and 0.256 for daily torpor, hibernation, and homeothermy, respectively. Here, time was measured by the proportion of branch lengths for which lineages are predicted to use a specific regime in the phylogeny averaged over all simulations.

Based on the ancestral state reconstruction, hibernation appears to have evolved four times in bats. Vespertilionidae and Molossidae both have taxa that use hibernation. Their most recent common ancestor may have used hibernation (logL = 0.533). The MRCA of Vespertilionids likely hibernated (logL = 0.850), and multiple internal nodes in Molossidae suggest a heterothermic ancestor. Our results suggest that hibernation evolved at least once in this group. Our data suggest that hibernation evolved independently at least three other times, in the species *Taphozous melanopogon* (Family Emballonuridae) and *Mystacina tuberculata* (Family Mystacinidae), and again in the clade comprised of Rhinopomatidae and Rhinolophidae. However, the node(s) at which hibernation arose in these groups remains ambiguous.

## 4 DISCUSSION

We found that the common ancestor of bats most likely used daily heterothermy and is very unlikely to have used hibernation. We also found that leptin evolution is not associated with the evolution of thermoregulatory regimes in bats. Twente and Twente (1964) hypothesized that the most recent common ancestor of bats was homeothermic and that heterothermy evolved secondarily as an adaptation to survive cold climates (Bieber & Ruf, 2009; Geiser & Turbill, 2009; Kortner & Geiser, 2000). However, daily heterothermy remains adaptive even at warmer temperatures because it increases energy savings and long‐term survival (Geiser & Stawski, 2011; Stawski & Geiser, 2012). Flexible use of torpor may have enhanced the ability of some bats to survive in warm and/or tropical climates, such as the environments likely encountered throughout the Eocene when bats likely originated (Amador et al., 2016; Czenze & Dunbar, 2017; Czenze, Brigham, Hickey, & Parsons, 2017b; Meredith et al., 2011; O'Leary et al., 2013; Simmons, 2005; Simmons & Geisler, 1998; Simmons, Seymour, Habersetzer, & Gunnell, 2008; Teeling, 2005).

Supporting our results, recent work estimating the ancestral thermoregulatory regimes of bats (Yuan et al., 2011) and evidence for the commonality of heterothermy in bats (Geiser & Stawski, 2011) suggests that heterothermy was the ancestral state for Chiroptera and that homeothermy was secondarily derived (Geiser & Stawski, 2011; Yuan et al., 2011). This scenario mirrors the hypothesis that the common ancestor of all mammals was heterothermic (Grigg et al., 2004; Lovegrove, 2012). However, until now, the question of which heterothermic regime was used by the ancestor of bats—hibernation or daily heterothermy—was unresolved. Here, we show evidence that the ancestor of bats was likely a daily heterotherm.

Consistent with the lack of evolutionary advantages that a hibernator would be expected to accrue during the early Eocene, a relatively warm time period characterized by widespread tropical and subtropical conditions, our results suggest that the common ancestor of bats did not hibernate (Humphries, Thomas, & Speakman, 2002). Although our results suggested a marginal likelihood that the ancestor of bats was a homeotherm, this seems unlikely based on previous studies (e.g., Geiser & Stawski, 2011; Yuan et al., 2011). Taken together with the high likelihood of daily heterothermy at this node, we argue that the most recent common ancestor of bats was a daily heterotherm. Our reconstruction therefore also indicates several reversals back to daily heterothermy. *Pteronotus davyi*,* Nyctimene major*,* Macroglossus minimus*, and *Syconyteris australis* all represent reversals back to daily heterothermy after their lineages evolved homeothermy. However, due to inconsistent methods for measuring *T*_b_ and the setting of arbitrary thresholds to determine a torpid state (Levesque et al., 2016), some of the bats categorized as homeothermic in our study may in fact be heterothermic. This may alter the interpretation of reversals back to heterothermy. Increased data collection following consistent operationalized definitions of torpor should be performed for more species and recollected for species currently identified as homeothermic. Our suspicion is that many bats thought to be endothermic are actually facultative homeotherms under some conditions (e.g., see Czenze & Dunbar, 2017). Future work should also focus on other species across the mammal phylogeny in order to reconstruct the ancestral states at deeper nodes.

Our analyses suggest that hibernation evolved approximately four times in Chiroptera—at the base of Vespertilionidae and Molossidae, in the species *Taphozous melanopogon* and *Mystacina tuberculata* from the families Emballonuridae and Mystacinidae, and in the clade comprised of Rhinolophidae and Rhinopomatidae. Our analyses also suggest that the MRCA of Rhinolophidae used hibernation; however, it is unclear if this is derived or ancestral. Conservatively, we suggest that hibernation arose at least once in this group. Similarly, we suggest that hibernation arose at least once in the clade comprised of Vespertilionids and Molossids. We found no evidence for reversals in the hibernation phenotype—no lineages that lost and subsequently regained the ability to hibernate.

**Figure 4:**
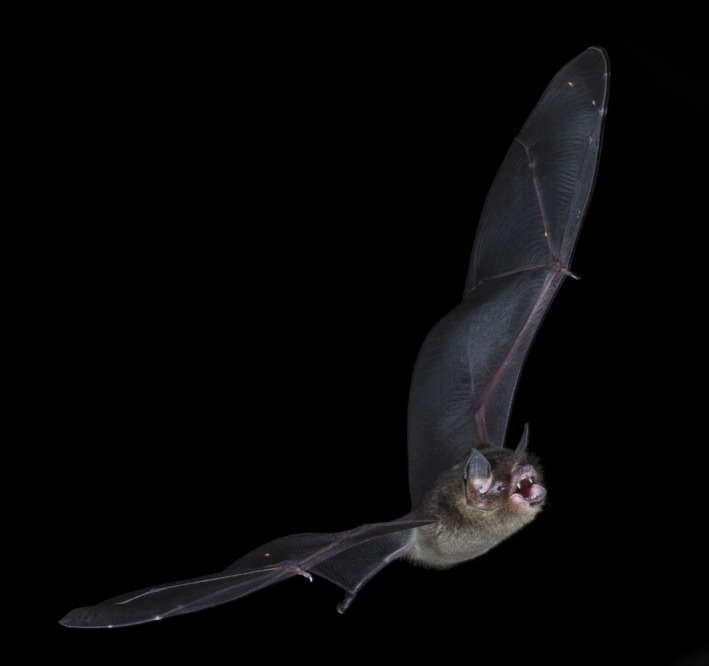
*Pteronotus parnellii* photograph taken by Brock and Sherri Fenton in a cave in the western end of Cuba

Significant evidence for positive selection of *lep* was detected in some lineages of Chiroptera, but this had no correlation with the thermoregulatory regimes of those lineages (Figure 1b). Compared to results from Yuan et al. (2011), our dataset included 11 additional bat taxa, and the thermoregulatory regimes of *Carollia brevicauda* and *Pteronotus parnellii* (Figure 4) were reclassified to be consistent with the literature (Avila‐Flores & Medellín, 2004; Bonaccorso, Arends, & Genoud, 1992). Leptin evolution is impacted by pleiotropic effects on other physiological and developmental processes beyond thermoregulation. Leptin can effect thyroid function (Ghamari‐Langroudi et al., 2010) and bone development (Crespi & Denver, 2016), induction of mitosis (Gat‐Yablonski & Phillip, 2008), and immune and stress responses (Ahima & Osei, 2004; Procaccini, Lourenco, Matarese, & La, 2009). The potential for positive selection of leptin for alternative traits (Carey, Andrews, & Martin, 2003; Jastroch et al., 2016; Yang et al., 2008) makes it difficult to find correlations between positive selection of *lep* and a singular function. Therefore, in retrospect it is perhaps not surprising that we found no evidence for a tight correlation between leptin and chiropteran thermoregulatory regimes, and also found no correlation between leptin selection and diet despite the known influence of high‐fat diets on leptin functioning (Frederich et al., 1995; Koch et al., 2014).

Complex genomic mechanisms and associated physiological alterations to organ systems and physiological functions across species with different thermoregulatory regimes suggest that the evolution of thermoregulatory regimes may intrinsically have no correlation with positive selection of a single gene (Andrews, 2004; Grabek et al., 2011; Hindle, Grabek, & Epperson, 2014; Morin & Storey, 2009; Villanueva‐Cañas, Faherty, Yoder, & Albà, 2014). A non‐synonymous substitution at codon site 91 in exon 3, found here to be under positive selection, was previously inferred to cause a functional difference in hibernating bats compared to homeothermic bats (He et al., 2010). However, we found that this substitution in the sequence of the hibernating bat is shared with *Homo sapiens*, a homeothermic species. Therefore, a direct relationship to thermoregulatory regime and sequence variation cannot be made for this site. Recent evidence suggests that thermoregulatory regimes are mostly influenced by the regulation of gene expression, rather than the sequence specificity of protein‐coding genes (Geiser & Stawski, 2011; Grabek, Martin, & Hindle, 2015; Morin & Storey, 2009; Schwartz, Hampton, & Andrews, 2013; Yan, Barnes, Kohl, & Marr, 2008).

In our ancestral state reconstruction, zero direct transitions occurred between hibernation and homeothermy. In this model, we assumed a priori that character states are not ordered. Therefore, the lack of transitions between hibernation and homeothermy is not an artifact of the model. This suggests that thermoregulatory regimes may be better represented as an ordered trait (with daily torpor as a necessary intermediate between homeothermy and hibernation) rather than as an unordered trait. Recent evidence suggests that heterothermy exists along a continuum (Boyles et al., 2013; Dunbar & Brigham, 2010; Lovegrove, 2012; Wilz & Heldmaier, 2000). If true, this suggests that there is an inherent order to the evolution of thermoregulatory regimes, which our results indicate. Our results suggest that future research on mammalian thermoregulation should treat thermoregulatory regimes as an ordered and possibly continuous trait.

The genomics underlying thermoregulation in mammals remains largely unclear. Future research should aim to sequence whole genomes of mammals that vary in thermoregulatory regime. Comparing these data in a phylogenetic framework would enable a more complete understanding of the genomic components involved in the evolution of thermoregulatory regimes. Our results revealed that leptin does not appear to be directly involved in the evolution of thermoregulatory regimes but many candidate genes including LEPR (Rezai‐Zadeh et al., 2014), MEF2 (Tessier & Storey, 2010), and G0S2 (Jessen et al., 2016) have yet to be examined in a similar framework. Such studies will reveal the importance these candidate genes have in the evolution of thermoregulation across diverse taxa.

## CONFLICT OF INTEREST

None declared.

## AUTHOR CONTRIBUTIONS

M.E.L, F.T.B., and N.B.S. contributed to the concept and design of project. M.E.L. performed laboratory work, data collection, and analyses, and drafted the manuscript. All authors contributed to manuscript preparation and approval of final draft.

## DATA ACCESSIBILITY

Sequence data are available on GENBANK (https://www.ncbi.nlm.nih.gov/genbank/) with accession numbers listed in Table 1. Log likelihoods for the thermoregulatory regime at each internal node in the phylogeny under the Mkn model are available in Supporting Information Table S1. All other data can be accessed through Dryad or by contacting Margaret Lazzeroni.
